# Evaluation of fertility toxicity and embryo-fetal developmental toxicity profile of Ayush AG, a compound herbo-mineral Ayurvedic formulation

**DOI:** 10.1016/j.jaim.2025.101154

**Published:** 2025-06-28

**Authors:** Shrirang B. Jamadagni, Mukul Tambe, M. Srinivasan, Sudesh Gaidhani, Goli Penchala Prasad, Sudhir Matte

**Affiliations:** a Regional Ayurveda Research Institute, Pune (Peripheral Unit of Central Council for Research in Ayurvedic Sciences, New Delhi, Ministry of AYUSH, Govt. of India) Nehru Garden, Gandhi Bhavan Road, Kothrud, Pune, 411038, India; b National Ayurveda Research Institute of Panchakarma, Cheruthuruthi, (Peripheral Unit of Central Council for Research in Ayurvedic Sciences, New Delhi, Ministry of AYUSH, Govt. of India) Thrissur District, Kerala, 679531, India; c National Institute of Indian Medicine Heritage, Hyderabad (Peripheral Unit of Central Council for Research in Ayurvedic Sciences, New Delhi, Ministry of AYUSH, Govt. of India), Survey No. 314, Revenue Board Colony, Gaddiannaram, Hyderabad, 500036, India

**Keywords:** Embryo-fetal developmental toxicity, Ayush AG, Rats, Reproductive toxicity

## Abstract

**Background:**

It is essential to generate the reproduction toxicity data of any drug that will be used in pregnancy or during the reproductive phase of life. Ayush AG, a compound herbo-mineral Ayurvedic formulation that is developed for promoting the health of the fetus and mother, was tested to assess its reproductive toxicity profile as per the standard guidelines.

**Materials and method:**

In the fertility toxicity study, Ayush AG, the test drug was administered in three graded doses to Wistar rats (40 male and 40 female) during the premating, mating period, gestation, and lactation periods. Parameters such as estrous cycle, body weight, feed consumption, hematology, serum biochemistry, pup body weight, hematology and biochemistry, histopathology of reproductive organs and reproductive indices were studied. In the embryo-fetal developmental toxicity study, the test drug was administered from day 7 to day 9 of the gestation period in 18 pregnant rats and sacrificed on gestation day 20. Parameters such as body weight during gestation, uterus weight, fetus and placenta weight, gross examination of fetuses for sex, external and visceral anomalies were studied.

**Results:**

In the fertility toxicity study, no adverse effect on mating behaviour, estrus cycle, conception rate, body weight, hematological and biochemical parameters, pup growth, or histopathology was found at dose level of 750 mg/kg of Ayush AG. No teratogenic changes were observed in the fetuses in the embryo-fetal developmental toxicity study at dose level of 750 mg/kg.

**Conclusion:**

Ayush AG, the compound herbo-mineral Ayurvedic formulation, has been demonstrated to be safe at the dose of 750 mg/kg in fertility toxicity and embryo-fetal developmental toxicity studies in Wistar rats.

## 1 Introduction

Preclinical toxicity testing of pharmaceutical products underwent strategic modifications following the Thalidomide tragedy [1]. Thalidomide, a promising candidate drug to cure morning sickness related to pregnancy, itself directly produced severe teratogenic effects in the fetuses [1]. Subsequent to the occurrence of the Thalidomide incident in the late 1960s, drug regulatory authorities and toxicologists adopted a cautious approach to assess the reproductive toxicity profile of prospective drug candidates [1,2]. Consequently, it was recommended that, irrespective of the toxicity profile observed during routine systemic toxicity studies, drug candidates must undergo stringent reproductive toxicity testing protocols in laboratory animals, developed through comprehensive consultation and validation across the globe [[1], [2], [3], [4], [5], [6]]. Pregnancy causes physiological alterations, which can affect the pharmacokinetics of drugs as well [7]. Pregnant women consuming prescription medications for therapeutic purposes pose a risk to their developing fetuses with significant structural and functional side effects due to altered pharmacokinetics [8,9]. In the Indian scenario, the availability of over-the-counter (OTC) drugs and self-medication act as significant factors leading to the consumption of drugs during pregnancy, which may expose the developing fetus to teratogens. Additionally, it is also reported that pregnant women in particular geographical areas would instead treat pregnancy-related symptoms using oils, herbal remedies, or herbs as opposed to prescription drugs [[9], [10], [11]]. Experts have drawn attention to the fact that due to the unavailability of sufficient scientific data, it would be potentially risky to believe that over-the-counter medicines, which are marketed as herbal and healthy for non-pregnant women, are safe for use during pregnancy [12].

As a result, the investigation into the toxicity and safety of Ayurvedic medications and polyherbal combinations is necessary.

The concept of *"Garbhini Paricharya,"* or prenatal care from conception to childbirth, is outlined in Ayurveda. Antenatal care in Ayurveda includes *Aushadhi* (medicines), *Vihara* (lifestyle), and *Aahar* (dietary plans). Ayurvedic polyherbal formulation Ayush AG was developed by the New Delhi-based Central Council for Research in Ayurvedic Sciences (CCRAS) [11,13]. The formulation was developed to exploit the potential benefits of Ayurvedic herbs for enhancing the health of pregnant women and their offspring. Ayush AG consists of *Emblica officinalis* Gaertn (Amalaki/Awala), *Asparagus racemosus* Wild (Shatavari), *Withania somnifera* (L.) Dunal (Ashwagandha), *Mandur Bhasma* (calcined iron rust), along with *Muktashukti Bhasma* (calcined oyster pearl shell) [11]. It is recommended that the general health of pregnant women and fetal growth be promoted for the healthy development of the newborn [11]. Although the systemic toxicity and safety-related data of the individual ingredients, such as *Emblica officinalis, Withania somnifera, and Lauha Bhasma*, are available, the reproduction toxicity profile of the Ayush AG formulation is not available [[14], [15], [16]]. Since the drug is intended for therapeutic use in pregnant women, it becomes imperative to assess its potential to cause reproduction toxicity and teratogenicity using preclinical toxicity protocol as per the standard *in vivo* tests mentioned in regulatory guidelines.

Since Ayush AG is intended to be used in pregnant women, it becomes imperative to assess its potential to cause fertility toxicity and teratogenicity using preclinical toxicity studies as per the standard methodologies mentioned in various guidelines. However, no such preclinical data regarding the Ayush AG is available at present; hence, these particular studies were carried out.

## 2 Materials and methods

### 2.1 Test drug

Ayush AG was manufactured by Indian Medicines Pharmaceutical Corporation, Mohan, Almora, Uttarakhand, with batch number AYU 17 and an expiry date of 05/24. The drug analysis was performed in compliance with the Ayurvedic Pharmacopoeia of India (API) parameters at the Delhi Testing House laboratory located at Azadpur, Delhi, for physicochemical properties, pesticide residue levels, heavy metal residue levels, aflatoxin and microbial contamination levels. The analysis report showed that the pesticide residue levels, heavy metal residue levels, aflatoxin and microbial contamination levels were within acceptable limits. Other physicochemical parameters, such as total ash, acid-insoluble ash, disintegration time, hardness, friability, dissolution time, pH, and loss on drying, were within the pharmacopoeia limits. The formulation for administration to the animals was prepared daily before the dosing by weighing the required amount of the drug, crushing it in a mortar and pestle and suspending it in 0.5 % carboxymethylcellulose (CMC) solution. The final formulations had concentrations of 75 mg/mL for the High dose (750 mg/kg) group, 50 mg/ml for the Mid dose (500 mg/kg) and 25 mg/mL for the Low dose (250 mg/kg).

### 2.2 *In vivo* experimentation and animal welfare

The *in vivo* studies were conducted at a CCSEA-registered animal housing and breeding facility. The Institutional Animal Ethics Committee (IAEC) provided the necessary ethical permissions under approval numbers IAEC/2019_2/16 and IAEC/2019_3/03 for the conduct of Segment 1 and Segment 2 studies. Segment 1 study is performed to assess the adverse effects of drug exposure on fertility, estrus cycle, mating performance, conception rate, growth of the fetuses and growth of the pups, along with impact on spermatogenesis in male animals. Segment 2 study is performed to assess the adverse effects of drug exposure during the embryo-fetal development phase. Adult (9–10 weeks old) male and female (nulliparous) Wistar rats (Crl: WI Charles River, USA strain) that were free of disease were acquired from the institute's animal breeding facility.

Animals were acclimated for seven days. The body weight of all the animals was recorded on receipt, and the health examination was performed during the acclimatisation period. Relative humidity and temperature were kept at 60–70% and 22 + 10 °C, respectively, and lighting was adjusted to provide about 12 h of light and 12 h of darkness. Each animal was kept in a 43 x 27 × 15 cm polypropylene cage having lid of stainless-steel and bedding made of corncob. All the animals were provided *ad libitum* regular rodent pellet diet (Altromin International, Germany). Purified and deionised water was made available all the time using polypropylene bottles with steel nozzles. The maximum dose volume administered was 10ml/kg body weight. The oral route is the intended route of drug administration in humans. The test drug was orally administered using a gavage needle of 16 gauze.

#### 2.2.1 Fertility toxicity study

Based on their body weight, 40 male and 40 female rats (9–10 weeks old) were selected and randomly divided into four groups to achieve approximately similar mean body weights (SD < 20%) across all groups. Animals were identified by making tail markings using permanent markers. OECD 421, ICH S (5)R(2) and Schedule Y of Drugs and Cosmetic Act 1940 guidelines on reproduction and developmental toxicity screening were used as a reference document for performing the procedures and study design [[17], [18], [19]]. The test drug was administered to both male and female rats in the fertility toxicity study for 14 consecutive days before mating, during mating, and during gestation until lactation day 13. The total dosing period for male rats was 28 days.

Based on the results of acute and dose range finding toxicity studies, dose levels of Ayush AG tablets were selected as 250 (Low dose), 500 (Mid dose), and 750 (High dose) mg/kg + Vehicle Control (0.5%w/v CMC). Two animals per cage were housed in the premating period, and dams were housed individually after the mating.

All animals were observed daily for general clinical signs, morbidity and mortality twice a day at the same time. Body weights of each animal were recorded at the start of the study (day 1) and then at weekly intervals till mating, and weight gain was calculated and expressed as g/100 g body weight. The weekly feed consumption of rats was calculated and presented in g/100 g body weight/day. During the gestation period, measurements were made of the dam's body weight and feed consumption on day 0 (the day of mating), 3, 6, 9, 12, 15, and 18. After the littering, the body weight of the dams and pups, along with the feed consumption of the dams, were measured during the lactation period from day 1 (day of littering), 4, 8, 12, and 14.

Various reproductive performance-related indices, such as the mating index (%), female fertility index (%), and gestation index (%) of the males and females, were calculated.

Blood samples of dams and selected pups were collected for hematology in Ethylene Di-amine Tetra Acetate (EDTA) coated vials, whereas centrifuge tubes without anticoagulant were used for biochemical analysis. Sodium, potassium, chloride, total bilirubin, urea, cholesterol, triglycerides, albumin, alkaline phosphatase, glucose, total protein, ALT, AST, and serum creatinine were evaluated. HCT (%), platelet (PLT) count, WBC count, RBC count, mean corpuscular hemoglobin concentration (MCHC), hemoglobin (Hb), mean corpuscular volume (MCV) and mean corpuscular hemoglobin (MCH) were measured. Blood thyroxine (T4) and thyroid stimulating hormone (TSH) levels were also measured using the ELISA method (Krishgen Biosystems, Mumbai) from five randomly selected pups of each group.

#### 2.2.2 Embryo-fetal developmental toxicity study

A total of 18 pregnant females of 9–10 weeks of age were selected based on their body weight. The vehicle control group contained six females, the disease control Aspirin group included six females, and the Ayush AG group contained six females. The number of animals in the study was 6 as per the ICHS5 (R3) guideline [18]. In this study, the test drug was administered from gestation day 7–19, and animals were sacrificed one day before littering, i.e. on gestation day 20. The Ayush AG dose level was 750 mg/kg. The disease control group was dosed with Aspirin (250 mg/kg). The vehicle control group was dosed with CMC (0.5%w/v) based on the body weight of the animals. During the gestation period, measurements were made of the dam's body weight on day 0 (the day of mating), 3, 6, 9, 12, 15, and 18. All animals were observed daily for general clinical signs, morbidity and mortality twice a day at the same time.

### 2.3 Euthanasia and postmortem examination

#### 2.3.1 Fertility toxicity study

All the male animals were sacrificed after 28 days of drug treatment (including 14 days of pre-mating and mating period) using the carbon dioxide asphyxiation method of euthanasia method, and a detailed postmortem examination of each animal was done to record the gross pathological changes if any and organ collection. The weights of adrenals (2), kidneys (2), heart, liver, testes (2), epididymis, prostate, seminal vesicle, spleen, brain, and thymus were recorded. Testes were fixed in Modified Davidson's fixative for initial 24 h, followed by a 10% neutral buffered formalin solution. The prostate, epididymis, seminal vesicles, and coagulation glands were preserved in a 10% neutral buffered formalin solution.

All the dams, along with their pups, were sacrificed after lactation day 14 using carbon dioxide euthanasia methods, and a detailed postmortem examination of each pup and dam was performed to record the gross pathological changes, if any. Each dam's weights of adrenals (2), kidneys (2), heart, liver, ovaries (2), uterus, spleen, brain, and thymus were recorded.

Samples of testes, epididymis, ovaries, liver and seminal vesicles from the control and high-dose groups were processed for preparation of 5-μm thick sections. The sufficiently fixed tissue samples were cut and trimmed in the desired shape and size, immersed in ascending grades of alcohol followed by xylene, and then embedded in paraffin to prepare the blocks. The blocks were sectioned on a microtome to 5-μm thickness to prepare sections on the glass slides. The sections on the slides were stained with routine Hematoxylin and Eosin (H&E) staining protocol and subjected to histopathology evaluation by a pathologist.

#### 2.3.2 Embryo-fetal developmental toxicity study

All the females were sacrificed on gestation day 20 using the carbon dioxide euthanasia method, and a detailed post-mortem examination of each animal was done to record the gross pathological changes, if any. Each dam's post-mortem examination was performed to record the weight of the gravid uterus and its gross examination. The uteri were further observed to measure the number of live and dead fetuses, implantation sites, corpora lutea (CL), and early and late resorptions. The weight of each fetus and its placenta was recorded, and the crown-rump length of each fetus was measured. Further, fetuses were subjected to external observation and visceral examination to record developmental anomaly/malformation due to the teratological effect of the test drug.

### 2.4 Statistical analysis

The one-way ANOVA was used to statistically analyze the means of body weights, feed consumption, relative organ weights, hematology values, serum biochemistry parameters, pup developmental data, litter data, embryo-fetal developmental indices such as gravid uterus weight, placental and fetal weight, implantation sites and corpora lutea numbers, and crown-rump length. Tukey's test was carried out after a one-way ANOVA using the SPSS Statistics 16.0 software.

## 3 Results

### 3.1 Fertility toxicity study

The Ayush AG treatment did not show any clinical signs and mortality in any group during the study period.

#### 3.1.1 Male

No effect was observed on body weight gain and feed consumption due to Ayush AG treatment when compared to control groups in males, as shown in the table below (Table 1). Relative liver weight in High Dose was significantly increased compared to but was found to be within biological limits. No significant difference in relative weights of other organs was observed when compared between the Vehicle Control and treatment groups (Table 4). Hematology and serum biochemistry parameters values showed significant group-wise changes in values of hematocrit, platelet, total leukocyte, lymphocyte, monocyte, neutrophil, AST, total protein, ALT, alkaline phosphatase, albumin and globulin as compared to the Vehicle Control. However, the observed changes were within biological limits and were considered biologically non-significant [20] (Table 5, Table 6). Thus, Ayush AG treatment did not adversely affect the males.

**Table 1:** Body weight gain and feed consumption pre-mating -males and females (n = 10).

Group	Body weight gain(%)				Feed consumption (Grams/100 g b.wt./day)			
	Male		Female		Male		Female	
	Week 1	Week 2	Week 1	Week 2	Week 1	Week 2	Week 1	Week 2
Vehicle control	5.08 ± 5.196	11.06 ± 5.72	0.45 ± 6.7	8.18 ± 2.46	5.08 ± 5.19	11.06 ± 5.72	0.45 ± 6.7	8.18 ± 2.46
Low dose	12.21 ± 15.23	12.11 ± 6.5	3.99 ± 6.14	6.85 ± 9.46	12.21 ± 15.23	12.11 ± 6.5	3.99 ± 6.14	6.85 ± 9.46
Mid dose	4.37 ± 3.59	7.98 ± 5.2	1.71 ± 3.94	4.57 ± 4.84	4.37 ± 3.59	7.98 ± 5.2	1.71 ± 3.94	4.57 ± 4.84
High dose	4.67 ± 3.19	10.29 ± 4.39	2.17 ± 3.4	6.64 ± 7.86	4.67 ± 3.19	10.29 ± 4.39	2.17 ± 3.4	6.64 ± 7.86

Values are expressed as Mean ± SD.

**Table 2:** Gestation period body weight gain and feed consumption.

	Body weight gain(%)				Feed consumption (Grams/100 g b.wt./day)			
Gestation Day	Vehicle control	Low Dose	Mid Dose	High Dose	Vehicle control	Low Dose	Mid Dose	High Dose
GD3	5.99 ± 2.44	6.59 ± 8.45	3.21 ± 2.11	3.61 ± 3.02	2.41 ± 0.22	2.40 ± 0.32	4.68 ± 6.25	2.50 ± 0.18
GD6	10.35 ± 6.25	15.55 ± 12.96	4.82 ± 6.95	12.71 ± 8.07	2.79 ± 0.45	2.72 ± 0.28	5.89 ± 8.18	2.78 ± 0.25
GD9	21.65 ± 8.75	20.5 ± 16.15	5.57 ± 12.04	12.13 ± 8.52	3.15 ± 0.39	3.27 ± 0.39	7.55 ± 10.91	3.47 ± 0.33
GD12	24.73 ± 8.4	23.47 ± 8.69	11.56 ± 13.04	20.28 ± 7.5	3.74 ± 0.47	3.86 ± 0.30	8.99 ± 13.38	3.60 ± 1.29
GD15	31.61 ± 8.63	31.13 ± 8.74	26.7 ± 7.09	30.75 ± 6.78	4.26 ± 0.57	4.35 ± 0.40	10.25 ± 16.58	4.00 ± 1.42
GD18	45.58 ± 12.39	43.93 ± 11.74	37.03 ± 6.61	42.18 ± 9.47	4.49 ± 0.47	4.65 ± 0.24	11.52 ± 19.14	4.28 ± 1.53
GD21	61.92 ± 13.20	58.22 ± 16.42	52.25 ± 3.51	56.84 ± 12.34	4.62 ± 0.53	4.89 ± 0.21	12.31 ± 21.09	4.36 ± 1.56

Values are expressed as Mean ± SD.

**Table 3:** Lactation period body weight gain and feed consumption of the dams and pup weight.

	Dam weight gain (%)				Feed consumption during the lactation period (Grams/100 g b.wt./day)			

Lactation day	Vehicle control	Low dose	Mid dose	High dose	Vehicle control	Low dose	Mid dose	High dose

LD4	−5.94 ± 9.37	−1.55 ± 2.27	−1.21 ± 4.06	−0.68 ± 7.97	1.44 ± 0.15	1.56 ± 0.14	1.76 ± 0.15	2.24 ± 0.15
LD8	−9.75 ± 12.26	−2.85 ± 3.6	−1.89 ± 5.42	−1.96 ± 8.77	1.48 ± 0.17	1.63 ± 0.18	1.88 ± 0.26	2.38 ± 0.24
LD12	−8.14 ± 10.79	−1.77 ± 4.57	−2.11 ± 4.43	−1.36 ± 11.42	1.77 ± 0.29	1.74 ± 0.21	2.21 ± 0.26	2.65 ± 0.21
LD14	−8.45 ± 12.13	−2±4.45	−1.1 ± 5.46	−0.83 ± 10.55	4.12 ± 1.16	4.73 ± 0.63	5.42 ± 0.62	5.04 ± 0.33

	Lactation period body weight of the pups (g)							
LD1	7.22 ± 1.21	6.4 ± 1	5.46 ± 1.06	6.67 ± 1.93				
LD4	11.9 ± 3.01	9.9 ± 1.29	9.16 ± 1.51	10.74 ± 3.54				
LD8	16.86 ± 3.79	15.31 ± 3.05	13.53 ± 2	15.06 ± 4.27				
LD12	22.75 ± 4.63	19.16 ± 3.28	17.78∗ ± 2.23	19.17 ± 4.31				
LD14	28.57 ± 6.91	22.11∗ ± 2.78	20.00∗ ± 1.96	22.95 ± 5.74				

Values are expressed as Mean ± SD.

∗ Mean difference is significantly higher with p < 0.05 as compared to the vehicle control group.

**Table 4:** Dam and male relative organ weights (% of body weight).

Group	Liver		Spleen		Kidney		Adrenal		Heart		Thymus		Brain		Prostate	Testes	Epididymis	Ovaries	Uterus
	Dam	Male	Dam	Male	Dam	Male	Dam	Male	Dam	Male	Dam	Male	Dam	Male	Male			Dam	
Vehicle control	4.88 ± 1.00	4.02 ± 0.47	0.28 ± 0.05	0.23 ± 0.05	0.71 ± 0.06	0.77 ± 0.08	0.05 ± 0.01	0.06 ± 0.05	0.40 ± 0.04	0.34 ± 0.05	0.11 ± 0.04	0.1 ±0.02	0.61 ± 0.06	0.45 ± 0.04	0.35 ± 0.1	0.83 ± 0.11	0.49 ±0.14	0.09 ± 0.03	0.21 ± 0.07
Low dose	4.75 ± 0.43	3.75 ± 0.45	0.28 ± 0.03	0.23 ± 0.02	0.69 ± 0.12	0.78 ± 0.08	0.06 ± 0.02	0.05 ± 0.04	0.38 ± 0.04	0.34 ± 0.04	0.11 ± 0.04	0.12 ±0.01	0.58 ± 0.08	0.43 ± 0.03	0.32 ± 0.08	0.79 ± 0.08	0.54 ±0.06	0.08 ± 0.02	0.23 ± 0.06
Middle dose	5.27 ± 0.74	4.06 ± 0.62	0.32 ± 0.06	0.21 ± 0.03	0.74 ± 0.09	0.78 ± 0.09	0.07 ± 0.03	0.05 ± 0.05	0.41 ± 0.05	0.32 ±0.05	0.11 ± 0.04	0.11 ±0.03	0.63 ± 0.07	0.44 ± 0.08	0.38 ± 0.1	0.84 ± 0.07	0.49 ±0.08	0.09 ± 0.02	0.21 ± 0.07
High dose	4.82 ± 0.63	3.29 ± 0.4	0.29 ± 0.06	0.2 ± 0.02	0.71 ± 0.07	0.7 ± 0.07	0.07 ± 0.03	0.03 ± 0.01	0.42 ± 0.04	0.3 ± 0.03	0.12 ± 0.03	0.09 ±0.02	0.62 ± 0.07	0.4 ± 0.05	0.29 ± 0.1	0.74 ± 0.06	0.47 ±0.05	0.09 ± 0.03	0.25 ± 0.10

Values are expressed as Mean ± SD.

**Table 5:** Hematological changes in male rats, female rats and pups.

	Male				Female				Pups			
Parameters	Vehicle control	Low dose	Middle dose	High dose	Vehicle control	Low dose	Middle dose	High dose	Vehicle control	Low dose	Middle dose	High dose
TEC (106/μL)	8.39 ± 0.6	8.3 ± 1.21	8.11 ± 0.96	8.03 ± 0.85	8.37 ± 0.27	7.55 ± 0.75	7.25∗ ± 0.9	8.25 ± 0.62	7.24 ± 0.81	7.84 ± 1.06	8.06 ± 0.59	8.48 ± 1.24
Hb (g/dL)	15.41 ± 0.93	15.26 ± 1.13	16.27 ± 0.6	15.15 ± 1.25	15.54± 0.78	15.63± 1.43	14.99± 0.95	14.69± 0.94	15.61 ± 1.48	14.37 ± 1.31	15.16± 1.76	15.1 ± 1.43
HCT (%)	44.71 ± 5.13	35.50∗ ± 3.22	45.31 ± 2.5	44.62 ± 2.69	47.76 ± 3.25	36.14∗ ± 3.73	46.28 ± 3.18	45.26 ± 3.5	45.03 ± 3.67	36.06∗ ± 2.89	42.84 ± 2.77	48.86 ± 4.5
MCV (fL)	52.78 ± 3.52	51.37 ± 2.92	55.28 ± 3.53	54.27 ± 3.59	56.28 ± 3	54.02 ± 4.91	53.33 ± 4.15	53.76 ± 4.43	51.17 ± 5.19	52.59 ± 3.91	53.47 ± 3.27	54.18 ± 4.51
MCH (pg)	19.32 ± 1.39	19.27 ± 1.32	19.85 ± 0.99	19.14 ± 0.9	18.97 ± 1.25	20.13 ± 0.72	19.58 ± 1.19	18.57 ± 1.08	18.85 ± 1.89	19.53 ± 1.3	18.53 ± 1.55	18.32 ± 1.59
MCHC (g/dL)	34.84 ± 3.36	34.9 ± 2.83	36.01 ± 2.66	34.67 ± 2.11	34.28 ± 3.57	36.57 ± 1.67	34.22 ± 1.52	34.99 ± 1.44	36.24 ± 4.75	35.35 ± 1.96	34.79 ± 2.2	30.98 ± 2.45
PLT (103/μL)	813.5 ± 81.63	683.29∗ ± 95.2	844.75 ± 105.81	817.03 ± 101.03	893.75 ± 36.98	705.93∗ ± 52.32	741.43∗ ± 134.27	726.48∗ ± 135.13	740 ± 65.19	685.18 ± 58.98	787.2 ± 79.58	602.60∗ ± 79.29
TLC (103/μL)	3.58 ± 0.55	4.36 ± 0.73	2.91∗ ± 0.34	3.41∗ ± 0.43	3.62 ± 1.37	4.06 ± 0.84	4.17 ± 3.05	3.31 ± 0.34	5.12 ± 1.2	3.76 ± 1.62	3.88 ± 1.26	4.12 ± 1.57
Lymphocyte (%)	76.29 ± 5.1	75.34 ± 5.19	70.18∗ ± 3.14	68.36∗ ± 5.29	74.24 ± 4.54	75.84 ± 5.49	73.78 ± 6.95	71.76 ± 3.95	69.56 ± 5.41	73.91 ± 5.29	72.18 ± 10.6	70.02 ± 8.25
Monocyte (%)	2.22 ± 0.88	2.25 ± 0.61	3.04∗ ± 0.62	3.10∗ ± 0.42	2.93 ± 0.67	2.18∗ ± 0.18	2.61 ± 0.42	2.7 ± 0.34	2.8 ± 0.37	2.72 ± 0.99	2.46 ± 0.64	2.76 ± 0.31
Eosinophil (%)	2.42 ± 1.46	2.71 ± 1.05	3.39 ± 0.66	1.81 ± 0.52	2.84 ± 1.09	3.26 ± 0.15	3.44 ± 0.38	1.78 ± 0.5	3.34 ± 1.09	3.27 ± 0.54	2.88 ± 0.43	3.52 ± 0.86
Neutrophil (%)	19.54 ± 3.69	25.00∗ ± 4.13	23.9 ± 3.28	25.41∗ ± 4.64	20.76 ± 3.21	24.24 ± 2.56	26.77∗ ± 3.34	22.53 ± 3.54	25.06 ± 4.78	21.74 ± 6.22	22.48 ± 9.86	23.8 ± 8.78
Basophil (%)	0.4 ± 0.51	0.3 ± 0.48	0.2 ± 0.42	0.1 ± 0.31	0.33 ± 0.5	0.2± 0.42	0	0	0	0.13 ± 0.35	0	0
Reticulocyte (%)	3.05 ± 0.97	3.17 ± 0.82	3.085 ± 0.8	2.57 ± 0.92	3.18 ± 0.51	2.74 ± 0.38	2.61∗ ± 0.37	2.88 ± 0.25	3.02 ± 0.3	2.26 ± 0.52	2.74 ± 0.27	2.56 ± 0.74

Values are expressed as Mean ± SD.

∗ Mean difference is significantly higher with p < 0.05 as compared to the vehicle control group.

**Table 6:** Biochemical changes in male rats, female rats and pups.

	Male				Female				Pups			
Parameters	Vehicle control	Low dose	Middle dose	High dose	Vehicle control	Low dose	Middle dose	High dose	Vehicle control	Low dose	Middle dose	High dose
ALKP (U/L)	170.82 ± 17.6	150.52 ± 28.8	116.35∗ ± 31.78	126.34∗ ± 34.49	82.22 ± 10.38	77.39 ± 3.8	88.89 ± 17.29	87.62 ± 13.12	197.14 4.89	175.33 ± 16.71	132.84 ± 16.34	133.61 ± 11.47
Total bilirubin (mg/dL)	0.06 ± 0.03	0.09 ± 0.03	0.04 ± 0.02	0.05 ± 0.03	0.11 ± 0.11	0.07 ± 0.03	0.03∗ ± 0.02	0.05 ± 0.02	0.1 ± 0.03	0.84∗ ± 0.19	0.09 ± 0.06	0.07 ± 0.04
Total protein (g/dl)	9.73 ± 0.73	7.77∗ ±1.8	10.07∗ ±0.42	9.45∗ ±1.64	9.92 ± 0.88	6.34∗ ± 0.92	6.68∗ ± 1.11	9.27 ± 1.28	6.32 ± 0.76	11.09 ± 6.04	7.14 ± 0.5	6.84 ± 0.46
Albumin (g/dl)	5.97 ± 0.45	3.69∗ ± 0.45	4.14∗ ± 0.17	4.30∗ ±0.46	6.49 ± 1.24	4.13∗ ± 0.5	3.52∗ ± 0.76	4.15∗ ± 0.52	2.3 ± 0.45	7.56 ± 5.55	4.38 ± 0.44	4.09 ± 0.83
Globulin (g/dl)	3.93 ± 0.55	2.19∗ ± 0.35	1.97∗ ± 0.39	2.19∗ ± 0.5	4.59 ± 1.3	2.04∗ ± 0.52	2.37∗ ± 0.31	2.68∗ ± 0.83	4.01 ± 0.35	3.53 ± 0.6	2.76∗ ± 0.4	2.93∗ ± 0.42
A: G ratio	1.73 ± 0.3	1.74 ± 0.39	2.20 ± 0.55	2.07 ± 0.59	1.67 ± 0.18	2.16 ± 0.64	1.5 ± 0.35	1.68 ± 0.55	1.77 ± 0.22	2.03 ± 1.19	1.61 ± 0.3	1.44 ± 0.49
AST (U/L)	86.62 ± 7.55	68.05∗ ± 12.58	98.09 ± 6.61	79.46 ± 15.39	82.22 ± 10.38	77.39 ± 3.8	88.89 ± 17.29	87.62 ± 13.12	102.6 ± 6.04	94.5 ± 7.36	92.62 ± 4.54	97.04 ± 17.91
ALT (U/L)	34.77 ± 3.86	26.23∗ ± 4.89	29.92 ± 3.27	34.77 ± 3.86	36.46 ± 5.03	31.63 ± 1.97	32.35 ± 5.19	34.05 ± 4.59	39.83 ± 4.63	35.59 ± 11.23	38.49 ± 5.57	43.22 ± 3.91
Creatinine (mg/dL)	0.49 ± 0.08	0.43 ± 0.07	0.46 ± 0.05	0.43 ± 0.09	0.53 ± 0.14	0.43 ± 0.1	0.49 ± 0.09	0.46 ± 0.13	0.58 ± 0.05	0.5 ± 0.07	0.46 ± 0.17	0.5 ± 0.13
Glucose (mg/dL)	112.47 ± 8.02	119.97 ± 12.08	112.69 ± 14.03	104.53 ± 15.92	119.18 ± 14.28	130.01 ± 19.72	120.15 ± 27.3	131.48 ± 14.04	88.4 ± 20.13	82.93 ± 28.53	133.33 ± 27.14	127.22 ± 27.54
Sodium (mmol/L)	145.06 ± 1.1	142.05 ± 9.3	141.15 ± 2.73	148.16 ± 5.97	145.32 ± 1.16±	143.43 ± 7.04	144.87 ± 4.07	146.65 ± 4.06	144.12 ± 2.3	135.58 ± 23.83	146.08 ± 4.69	141.62 ± 6.63
Potassium (mmol/L)	4.59 ± 0.39	4.1 ± 0.77	3.72 ± 0.55	4.06 ± 0.8	4.34 ± 0.67	4.23 ± 0.69	3.92 ± 0.5	3.98 ± 0.68	3.68 ± 0.62	2.97 ± 0.85	3.16 ± 0.08	3.45 ± 0.67
Phosphorus (mg/dL)	8.49 ± 2.21	9.95 ± 1.41	9.92 ± 0.96	8.81 ± 1.55	7.81 ± 1.98	9.74 ± 1.31	9.74 ± 0.87	8.4 ± 2.06	7.74 ± 0.94	17.21 ± 3.05	9.15 ± 1.54	6.54 ± 0.8
Calcium (mg/dL)	8.57 ± 0.98	8.4 ± 1.1	9.12 ± 1.73	9.78 ± 3.36	9.34 ± 1.35	9.56 ± 1.48	9.19 ± 1.21	9.49 ± 1.11	10.38 ± 0.89	12.8 ± 3.19	10.42 ± 1.29	10.32 ± 0.96
Triglycerids (mg/dL)	45.94 ± 3.73	42.88 ± 3.76	44.45 ± 3.41	43.77 ± 4.99	46.7 ± 2.15	45.26 ± 2.46	45.85 ± 2.44	39.10∗ ± 5.17	45.77 ± 4.52	111.94∗ ± 11.68	42.15 ± 3.82	43.64 ± 4.68
Total cholesterol (mg/dL)	85.78 ± 6.88	68.4 ± 16.96	86.73 ± 17.14	93.75 ± 4.47	85.61 ± 12.39	81.33 ± 22.75	88.33 ± 14.99	72.84 ± 8.35	65.7 ± 18.89	85.91 ± 19.83	56.27 ± 13.2	52.87 ± 7.18
LDL (mg/dL)	47.25 ± 36.24	74.67 ± 20.45	55.38 ± 5.27	47.54 ± 10.91	44.01 ± 15.89	79.51∗ ± 26.44	50.14 ± 11.07	29.88 ± 7.76	50.96 ± 12.1	71.46 ± 8.3	51.63 ± 26.95	15.2 ± 5.17
HDL (mg/dL)	34.46 ± 4.49	29.87 ± 2.8	32.00 ± 3.84	34.83 ± 5.37	37.24 ± 2.57	30.79∗ ± 2.13	31.02∗ ± 6.94	36.49 ± 4.73	31.58 ± 5.85	29.26 ± 4.43	27.93 ± 2.96	33.18 ± 5.27

Values are given as mean ± SD; Mean difference is significantly higher with p < 0.05 as compared to the vehicle control group.

#### 3.1.2 Females

In females, no significant difference was observed in feed consumption and body weight gain between the control and treatment groups during the first and second week of the pre-mating phase (Table 1). In all groups of females, there no abnormality in the length and pattern of the estrous cycle was observed. There was no significant difference in the body weight gain between the control and treatment groups during the nursing phase or during the gestation period (Table 2, Table 3). The relative organ weights of the treatment groups and Vehicle Control did not show any significant difference (Table 4).

Few hematological parameter values showed significant group-wise changes, such as Total Erythrocyte Count (TEC), hematocrit, platelet count, monocytes, and neutrophil and reticulocyte count compared to the Vehicle Control. However, the changes observed were within biological limits [20] and were not dose-dependent and considered biologically non-significant (Table 5).

Serum biochemistry values such as ALKP, total bilirubin, total protein, albumin, and globulin triglycerides showed significant group-wise changes compared to Vehicle Control. However, the changes observed were within biological limits [20] and not dose-dependent; hence, they were considered biologically non-significant (Table 6).

#### 3.1.3 Pups

Pup body weights from treatment groups on Lactation Day (LD) 12 and LD 14 showed significant changes compared to the Vehicle Control. Nevertheless, the results were within biological reference ranges and the changes were not dose-dependent and hence were considered biologically non-significant [21] (Table 3).

The pups' hematology and serum biochemistry markers also revealed similar biologically non-significant changes in a few parameters like hematocrit values, total bilirubin, globulin, and triglycerides [20] (Table 5, Table 6). Serum T4 and TSH values of pups showed no treatment-related changes (Table 7).

**Table 7:** Reproductive indices of the dams and males and indices of the pups.

	Group/Dose (mg/kg/day)	Vehicle control	Low Dose	Mid Dose	High dose
Reproductive indices of the dams and males	No. of females placed with males	10	10	10	10
	No. of females showing evidence of mating	8	10	9	10
	No. of females pregnant	8	10	9	10
	No. of females with live pups	8	10	9	10
	Mating index (%)	80	100	90	100
	Female fertility index (%)	100	100	100	100
Developmental indices of the pups	Mean No. of implantation	14	12.3	14.33	13.1
	Mean total number of pups	14	12.3	14.33	13.1
	Mean number of male pups	6.67	6.3	7.11	6.4
	Mean number of female pups	7.33	6.1	7.22	6.7
	Mean sex ratio (Male/Female pups)	1.1	1.26	1.41	0.99
	Mean No of pups survived on LD14	13.67	12.1	14.22	13
	Mean live birth index (%)	100	100	100	100
	T4 (thyroxine) ng/mL	25.48 ± 1.98	22.27 ± 1.45	22.46 ± 0.67	23.24 ± 0.82
	TSH (thyroid stimulating hormone) uIU/mL	0.87 ± 0.07	0.85 ± 0.06	0.81 ± 0.06	0.78 ± 0.05

Values are given as mean ± SD for Pup serum T4 and TSH levels (N = 5).

#### 3.1.4 Histopathology

**Fig. 1:**
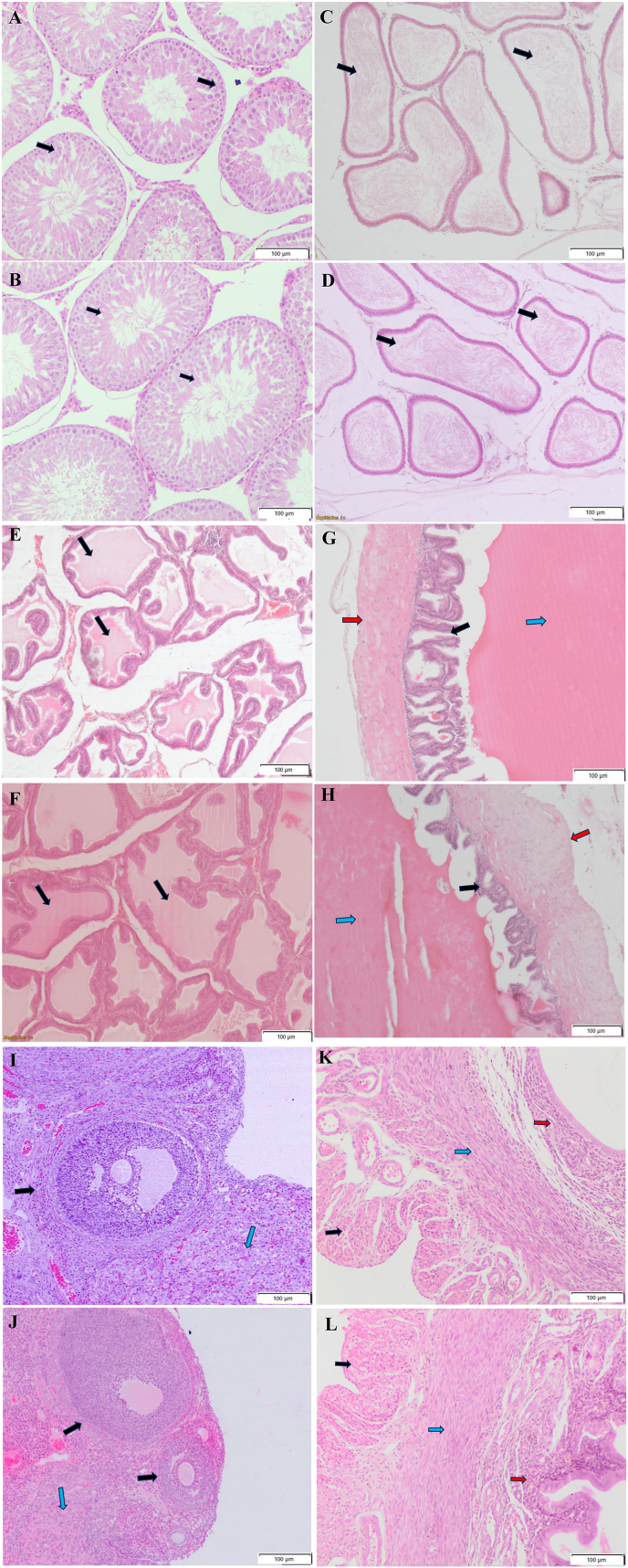
**A.** Testes; Vehicle Control, **B**. Testes; High Dose. Histomorphology shows no abnormality. Black Arrow: seminiferous tubules showing spermatogonial cells at various stages of development **C.** Epididymis; Vehicle Control, **D**. Epididymis; High Dose. Histomorphology shows no abnormality. Black arrow: lumen of epididymis with spermatozoa **E.** Prostate; Vehicle Control, **F.** Prostate; High Dose. Histomorphology shows no abnormality. Black Arrow: Acini with secretion **G.** Seminal vesicle; Vehicle Control, **H.** Seminal vesicle; High Dose. Histomorphology shows no abnormality. Black Arrow: Epithelium of seminal vesicle, Blue Arrow: lumen of seminal vesicle with secretion, Red Arrow: Smooth muscle layer **I.** Ovary; Vehicle Control, **J.** Ovary; High Dose. Histomorphology shows no abnormality. Black Arrow: ovarian follicles, Blue Arrow: corpus luteum **K.** Uterus; Vehicle Control, **L.** Uterus; High Dose. Histomorphology shows no abnormality. Black Arrow: perimetrium, Blue Arrow: myometrium, Red Arrow: Endometrium All image 10 x 10X. (For interpretation of the references to colour in this figure legend, the reader is referred to the Web version of this article.)

Histopathology evaluation of reproductive system organs like testes, epididymis, prostate, seminal vesicles, ovaries and uterus of the male and female parents showed no histomorphological changes or lesions due to administration of Ayush AG (Fig. 1: A- L).

#### 3.1.5 Reproductive indices

No drug treatment-related adverse effect on reproductive indices such as mating index (%), female fertility index (%), and gestation index (%) of the males and females, in males and females, was observed (Table 7). Further, there was no adverse effect of the drug treatment on postnatal developmental landmarks such as the mean sex ratio (Male: Female), the mean number of pups survived, mean live birth index, mean viability index on LD 14 (%) were within the biological reference limits [21] (Table 7).

### 3.2 Embryo-fetal developmental toxicity study

No group showed any abnormalities in clinical signs or death during the course of the study.

#### 3.2.1 Dam weight

No effect was observed on the body weight gain of the females when compared to the control, disease control and treatment groups during gestation (Table 8).

**Table 8:** Embryo-fetal developmental indices.

Sr. No.	Parameters	Vehicle control	Aspirin 250 mg/kg	Ayush AG		Gestation Day	Vehicle control	Aspirin 250 mg/kg	Ayush AG
1.	Mean placenta weight	0.44 ± 0.05	0.48 ± 0.07	0.57 ± 0.11	Gestation body weight (grams)	GD3	6.63 ± 3.75	8.79 ± 3.24	7.99 ± 4.09
2.	Mean fetal weight	1.26 ± 0.31	2.31 ± 0.4	2.87 ± 1.43		GD6	11.61 ± 3.29	15.99 ± 5.52	14.02 ± 5.69
3.	Mean crown rump length	1.95 ± 0.29	2.69 ± 0.3	2.91 ± 0.7		GD9	15.72 ± 3.96	18.53 ± 11.77	20.03 ± 7.55
4.	Dam weight at sacrifice	349.72 ± 28.6	340.92 ± 50.49	357.94 ± 21.88		GD12	21.92 ± 5.71	24.68 ± 13.38	27.73 ± 7.05
5.	Gravid uterus weight	43.46 ± 8.34	52.48 ± 19.36	57.34 ± 18.9		GD15	28.22 ± 7.24	34.59 ± 12.37	36.12 ± 8.82
6.	Corpus lutea	15.75 ± 2.22	12.20 ± 4.32	12.33 ± 3.88		GD18	39.02 ± 7.76	47.14 ± 14.53	45.12 ± 15.39
7.	Mean number of implantation sites	15.75 ± 2.22	12.20 ± 4.32	12.33 ± 3.88					
8.	Mean total live fetuses	15.75 ± 2.22	12.00 ± 4.53	12.17 ± 3.76					
9.	Mean number male fetuses	6.75 ± 0.96	6.00 ± 2.12	6.17 ± 2.4					
10.	Mean number female fetuses	9.00 ± 1.63	6.00 ± 2.55	6.00 ± 2.1					
11.	Sex ratio	1.35 ± 0.24	1.02 ± 0.22	1.05 ± 0.4					
12.	Live birth index	100 ± 0.0	97.8 ± 4.92	98.83 ± 2.86					

• Values are given as mean ± SD.

#### 3.2.2 Embryo-fetal developmental indices

To assess the teratogenicity of the test compound, various indices were evaluated, which included fetal weight, crown-rump length, implantation sites count, dam weight at sacrifice, gravid, uterus weight, corpora lutea, placental weight, total live fetuses, sex ratio of the fetuses, live birth index [2,18,22]. However, it was observed that administration of Ayush AG at 750 mg/kg did not induce any significant adverse effect on the various important embryo-fetal developmental indices. The details of the embryo-fetal developmental indices are presented in (Table 8).

#### 3.2.3 External and visceral anomalies

The test drug, Ayush AG, was administered in accordance with the test's principle during the organogenesis phase (GD 6–15) to observe the teratological effect in the form of various congenital anomalies exhibited during the visceral and external gross examination of the fetuses [2]. The details of the external anomalies/malformations are presented in Table 9. No significant test drug Ayush AG treatment-related increase in frequency or types of anomalies was observed. Further, the aspirin at the dose of 250 mg/kg induced anomalies were found to be as per the literature published [23].

**Table 9:** Embryo fetal developmental anomalies observed per group.

Anomaly	Vehicle control	Ayush AG	Asprin 250 mg/kg
Micrognathia	2 (63)	1 (73)	2 (61)
Hematoma in placenta	1 (63)	1 (73)	1 (61)
Domed head	2 (63)	1 (73)	6 (61)
Kidney hypoplasia	4 (63)	4 (73)	8 (61)
Bent forepaw	3 (63)	5 (73)	4 (61)
Open eyelid	2 (63)	4 (73)	3 (61)
Exopthalmus	0	0	2 (61)
Exencephaly	0	0	1 (61)

() indicate the total number of fetuses observed in the group.

## 4 Discussion

Ayurveda is believed to be the oldest system of medicine, offering a holistic approach to healthcare. Ayurvedic drugs can be in the form of compound herbo-mineral formulations, as in the case of Ayush AG. It is crucial to bridge the gap between conventional knowledge and contemporary research methodologies when it comes to scientifically validating Ayurvedic practices, including safety issues. Considering the unique properties of Ayurvedic drugs, modern research could incorporate Ayurvedic principles into standard study designs [24]. *Emblica officinalis* Gaertn, *Asparagus racemosus* Wild, *Withania somnifera* (L.) Dunal are the main herbal ingredients of Ayush AG, which are also being used in several other Ayurvedic formulations. Numerous tests on individual herbs in the Ayush AG, the herbo-mineral formulation demonstrate that they are safe for animal studies. For instance, several *Emblica officinalis* extracts are safe in preclinical animal studies [14,25]. *Withania somnifera* methanolic extract was studied in Wistar rats at the maximum dosage level of 2000 mg/kg and showed no toxicity to the mother or the fetus [15]. Similarly, testing the *Withania somnifera* root aqueous extract's mutagenicity and safety in different animal models revealed no mutagenic (genotoxic) effects at high concentrations [26]. In addition, different extracts of *Emblica officinalis* ameliorate male reproductive tissue damage induced by various toxins [27,28]. Moreover, Lauha Bhasma, the other mineral component of the Ayush AG, demonstrated safety at five times the therapeutic dose of 20.80 mg/kg [16]. The preclinical reproduction toxicity studies support the integration of Ayurveda into mainstream healthcare practices in promoting reproductive health by providing a scientific basis for the Ayurvedic drug's safety profile.

Reproductive toxicity can be described as any adverse effect that a chemical or xenobiotic can cause on any stage of the reproductive cycle, including the reproductive functions of males and females. This includes teratogenic effects on the embryo, fetus and newborn.

[29. The growing fetus is susceptible to xenobiotics, including drugs. The embryo-fetal developmental toxicity study, also known as teratogenicity or Segment 2 study, is a preclinical research model designed to assess the potential harm caused to a developing fetus by xenobiotics due to exposure during the organogenesis phase of the pregnancy [2,4]. CCRAS, being the developer of the proprietary Ayurvedic formulation Ayush AG, undertook the exercise to generate its reproduction toxicity data.

### 4.1 Fertility toxicity

The objective of the fertility toxicity study, also called the Segment-I study, is to identify the adverse effects the test drug may have on spermatogenesis, estrus cycle, functions of reproductive hormones, mating behaviour, conception, implantation, fatal development and lactation period development of the offspring [4,[17], [18], [19]]. Therefore, in the present study, male and female Wistar rats of reproductive age were treated with Ayush AG for two weeks before mating, through the gestation period and up to lactation day 14. During the study period, weekly body weight gain, feed consumption, esterous cycle, reproductive performance, body weights of pups, hematological, biochemical investigation, thyroid hormonal assay for the pups, and organ weight along with histopathological evaluation of reproductive organs were observed and analysed.

Body weight and feed consumption are important indicators of health. Feed intake and body weight can be directly affected by any adverse systemic effects from the administration of test drugs [4,29]. Treatment with Ayush AG had no adverse effects on feed intake or body weight gain during the mating period, implying that it had no effect on the normal physiology of both male and female animals.

Endocrine-disrupting chemicals (EDC) are substances that interfere with the body's endocrine systems. This interference can lead to pathologies that are generally dependent on hormonal dysregulation, such as infertility, congenital anomalies, abortions, developmental delays, diabetes, obesity, cancers, autoimmune disorders, fatty liver disease, etc. [[30], [31], [32], [33]]. The action of EDCs is mediated by the inhibition of important enzymes that maintain hormone homeostasis or by interfering with following receptors: i) nuclear receptors such as thyroid hormone receptor, retinoic acid receptors (RARs), retinoid X receptors (RXRs), vitamin D receptor, peroxisome proliferator-activated receptors (PPARs); ii) steroid hormone receptors such as androgen receptor (AR), estrogen receptor (ER), progesterone receptor (PR), glucocorticoid receptor (GR); and iii) xenoreceptors such as constitutive androstane receptor (CAR), pregnane X receptor (PXR), aryl hydrocarbon receptor (AhR) [34]. Metabolites from different plants have shown EDS-like activity in many studies by influencing various targets [[33], [34], [35]].

Esterus cyclicity depends on the balance of reproductive hormones like oestrogen, progesterone, FSH, and LH. In this study, the estrus cycle was found to have routine frequency and duration, i.e. 4-5 days. It was also observed that the duration of each stage of the estrus cycle was within the physiological limits [16,25]. EDS also could affect spermatogenesis, which is reflected in mating behaviour, conception rate, and histomorphology of reproductive organs by affecting testosterone and GnRH release and functions [36]. Any drug treatment-related dysregulation or imbalance in male reproductive hormones leads to abnormalities in spermatogenesis and changes in morphology and weights of gonads and accessory reproductive organs such as prostate and seminal vesicles [37]. However, the current investigation did not find a change in the weights of the male reproductive organs. Furthermore, the epididymis, prostate, testes and seminal vesicle histomorphology were not affected due to histopathological lesions, implying the absence of any adverse effect due to Ayush AG administration in male rats.

Gestational feed consumption and body weight gain are critical parameters for judging maternal and fetal health during pregnancy [28]. In case of toxicity due to the test drug, the body weight gain can get adversely affected due to changes in digestive physiology, leading to stunted or abnormal growth of the fetuses [[38], [39]]. In the present study, the treatment with Ayush AG did not show any adverse effect on the gestational body weight.

#### 4.1.1 Effect on the progeny

The reproductive toxicant can affect male and female-mediated fertility by influencing the quality of the gametes, i.e. sperms and ova [3,4,36]. The weight, sex ratio, and number of pups born were all unaffected by the test drug Ayush AG treatment, and no stillbirths were reported in the study. This implies that the male and female-mediated fertility and fetal development were unaffected due to Ayush AG administration.

Placental as well as lactational exposure to toxicants may lead to developmental defects in the offspring [37]. However, no statistically significant changes related to Ayush AG in body weight, hematology and biochemistry were observed. Thyroid hormone evaluations are recently included in the revised toxicity testing protocols by the OECD and the U.S. EPA as a marker for anticipating the neurodevelopmental alterations during the growth period of the offspring since the thyroid hormone is critically responsible for neuronal growth and development [40,41]. In the present study, TSH and T4 levels in pups' serum on LD 13 were measured and found to be within normal limits. These results imply that.

Ayush AG has no effect at the dose level of 750 mg/kg on post-natal development during the lactation period.

### 4.2 Embryo-fetal developmental toxicity

The fetus is vulnerable to the teratological effects of xenobiotics, particularly during organogenesis. The toxicant can induce the teratological effect on the developing embryo and fetus by either or a combination of multiple mechanisms such as endocrine disruption, specific receptor- or enzyme-mediated teratogenesis, folate antagonism, oxidative stress, neural crest cell disruption and vascular disruption and [42]. Embryo-fetal toxicity may result in early embryonic loss, reduced development and growth, fetal death, abortion, formation of anomalies and stunted development in the post-natal period of lactation [42]. Stage of development, tissue specificity, and influence of the dose are the critical factors responsible for the formation of the teratogenic effect due to the above-said mechanism [5,43]. The therapeutic use of the Ayush AG is the healthy growth of the fetus during gestation. Body weight and feed consumption are important indicators of health and toxicity and any adverse systemic effect due to test drug administration can directly impact feed consumption and body weight [14]. Moreover, the fetal weight and crown-rump length are well-accepted indicators of the retardation of the growth of the developing fetus [2]. Ayush AG treatment during gestation did not adversely affect fetal weight and crown-rump length. Similar results were observed in *Withania somnifera* methanolic extract-treated pregnant rats [15]. The placenta is a lifeline for the developing fetus in the uterus. It supplies nutrients from maternal blood, removes unwanted metabolic products from the fetus, and returns them to the maternal blood. The test drug toxicant can directly cause injury to the placenta, leading to the entry of toxicants into the fetal blood, or can disrupt the blood supply to the fetus, which ultimately produces a teratogenic effect in the fetuses [2,4,5,15,44]. Total live fetuses and the number of corpora lutea indicate the occurrence of early embryonic deaths due to test drug administration [2]. The present study did not find any statistically significant differences between the Ayush AG treated and the vehicle control group regarding the number of corpora lutea and total live fetuses, implying that the treatment had no impact on early embryonic development.

The fetal sex ratio may be affected due to the susceptibility of any particular sex to the toxic insult caused by the test drug, leading to the survival of opposite-sex fetuses, which is reflected in the sex ratio of the live fetuses [2]. The Ayush AG treatment did not change the sex ratio in the present study.

Various congenital Anomalies are formed due to the teratogenic effect of xenobiotic exposure. The embryo is particularly susceptible to toxicants, leading to malformations during the phase of organogenesis and neural crest differentiation, which is from days 6–15 of the rat embryo. Deformities are generally caused by mechanical factors and disturbances in blood supply from the placenta [2,5]. In the present study, it is noticeable that no significant increase in frequency and types of anomalies were observed in Ayush AG-treated animals as compared to the Vehicle control animals.

Overall findings from embryo-fetal developmental toxicity show that the test drug Ayush AG did not cause any adverse effect on the survivability of the embryos and fetuses. Also, it did not show any affinity towards foetuses of a particular sex or stage of development, leading to anomalies formation. The average placental weights and gross observation indicated the non-toxic effect of the Ayush AG on it. Thus, the Ayush AG treatment did not show any apparent embryo-fetal toxicity in the form of congenital anomalies, altered sex ratio, altered crown-rump length and fetal weights.

Hence, the Ayush AG dose of 750 mg/kg was found to be the No Observed Effect Level (NOEL) for the embryo-fetal toxicity study in rats.

## 5 Conclusion

Ayush AG, the compound herbo-mineral Ayurvedic formulation, has been demonstrated to be safe at the dose of 750 mg/kg in fertility toxicity and embryo-fetal developmental toxicity studies in Wistar rats. The research will help to alleviate the concerns of the herbo-mineral drug Ayush AG toxicity in pregnant and lactating women.

## Statement for author contributions in the main manuscript

The first and corresponding author, SBJ, contributed in the conceptualization of the study, preparation of methodology/study design, investigation, data curation, arranging resources, review and editing of the manuscript, visualization, and supervision.

MT, contributed in investigations, resources, formal analysis and writing of the original draft of the manuscript.

SM, contributed to carrying out formal analysis, investigations and resources. The fourth author, SG, contributed for conceptualization of the study, review and editing of the manuscript, visualization, project administration and project funding.

GPP, contributed in original draft writing, supervision, project administration and project funding.

SuM, contributed in original draft writing, its review and its edition.

## Declaration of generative AI in scientific writing

None.

## Funding sources

This work was funded by the intra-mural research scheme of the Central Council for Research in Ayurvedic Sciences, New Delhi, vide sanction letter 3–112/2019- CCRAS/Admin/10.13039/100016931IMR/3747 dated September 30, 2019.

## Conflict of interest

As per the ICMJE CoI disclosure, the authors declare no potential conflicts of interest with respect to the research, authorship, and/or publication of this article.
